# Supplementary material for: Phylogenetic Analysis and Genetic Structure of Schlegel’s Japanese Gecko (Gekko japonicus) from China Based on Mitochondrial DNA Sequences
Source: Genes (Basel). 2022 Dec 21;14(1):18. doi: 10.3390/genes14010018 (PMC9858143; doi:10.3390/genes14010018)
Supplement: Supplementary file 1 [file genes-14-00018-s001.zip › Table S1.pdf]

Table S1 325 Sample information of *G. japonicus* used in experiments in this study

Number of samples	Sampling site	Specimen number	Latitude and longitude
12	Anqing, Anhui	AHAQ01-12	30.52°N, 117.04°E
1	Lu'an, Anhui	AHLA01-20	32.56°N, 116.81°E
12	Wuhu, Anhui	AHWH01-05, AHWH07-10, AHWH12, AHWH14-15	31.34°N, 118.39°E
1	Nanping, Fujian	FJNP29	26.65°N, 118.18°E
3	Yong'an, Fujian	FJYA24-26	25.94°N, 117.36°E
2	Guiding, Guangzhou	GZGD01-02	26.34°N, 107.13°E
19	Guilin, Guangxi	GXGL1-19	25.16°N, 110.16°E
14	Longsheng, Guangxi	GXLS01-14	25.47°N, 110.01°E
5	Yongfu, Guangxi	GXYF01-05	24.59°N, 109.59°E
2	Yangshuo, Guangxi	GXYS01-02	24.78°N, 110.50°E
1	Huaxi, Guizhou	GZHX01	26.26°N, 106.40°E
3	Libo, Guizhou	GZLB01-03	25.42°N, 107.88°E
2	Longli, Guizhou	GZLL01-02	26.46°N, 106.99°E
1	Jingmen, Hubei	HBJM05	31.03°N, 112.20°E
13	Wuhan, Hubei	HBWH01-13	30.32°N, 114.21°E
13	Chengbu, Hunan	HNCB01-03, HNCB05-10, HHNCB12-15, HNCB17,	26.37°N, 110.30°E
8	Changde, Hunan	HNCD01-02, HNCD04-09	29.04°N, 111.69°E
19	Daoxian, Hunan	HNDX04-13, HNDX16-19, HNDX21-25	25.52°N, 111.57°E
15	Huaihua, Hunan	HNHH01, HNHH03, HNHH05, HNHH07-18	27.52°N, 109.95°E
16	Huitong, Human	HNHT01, HNHT03-17	26.86 °N, 109.71 °E
16	Huayuan,Hunan	HNHY01-05, HNHY07-15, HNHY18, HNHY19	28.35°N, 109.28°E
4	Shuangpai, Hunan	HNSP01-02, HNSP04-6, HNSP10-15	25.96°N, 111.63°E
5	Shaoyang, Hunan	HNSY01-05	27.14°N, 111.28°E
6	Tongdao,Hunan	HNTD01, HNTD03, HNTD05-08	26.16°N, 109.77°E
20	Xinhuang, Hunan	HNXH01-20	27.21°N, 109.18°E
12	Yueyang, Hunan	HNYY01-12	29.24°N, 113.08°E
12	Yongzhou, Hunan	HNYZ01-07, HNYZ09-10, HNYZ13-15	26.27°N, 111.35°E
4	Xinning, Hunan	HNXN01, HNXN03-04, HNXN06-08, HNXN12	26.44°N, 110.84°E

12	Lingshutan, Yongzhou, Hunan	YZLST1-12	26.22°N, 111.63°E
5	Hunan Yongzhou Qingtang Yueyan Forestry	QTYY01, QTYY02, QTYY04-06	25.49°N, 111.39°E
7	Zhuzhou, Hunan	HNZZ01-02, HNZZ04-08	27.50°N, 113.09°E
2	Rugao, Jiangsu	JSRG03, JSRG04	32.38°N, 120.58°E
24	Longnan, Jiangxi	JXLN01-24	24.54°N, 114.51°E
15	Yangxian, Shaanxi	SXYX04, SXYX09-11, SXYX13-17, SXYX19, SXYX21-22, SXYX25-27	33.23°N, 107.55°E
8	Hangzhou, Zhejiang	ZJHZ01, ZJHZ06, ZJHZ08-10, ZJHZ15-17	30.25°N, 120.20°E
11	Zhoushan, Zhejiang	ZJZS01-09, ZJZS11-12	29.96°N, 122.31°E
1	Lishui, Zhejiang	ZJLS15	28.45°N, 119.92°E
