# Supplementary material for: Phylogenetic Analysis and Genetic Structure of Schlegel’s Japanese Gecko (Gekko japonicus) from China Based on Mitochondrial DNA Sequences
Source: Genes (Basel). 2022 Dec 21;14(1):18. doi: 10.3390/genes14010018 (PMC9858143; doi:10.3390/genes14010018)
Supplement: Supplementary file 1 [file genes-14-00018-s001.zip › Table S2.pdf]

Table S2 Species sources and accession numbers of *COI* gene in *Gekko* downloaded from GenBank

Species	GenBank accession numbers
G. auriverrucosus	EU417716; EU417717; EU417718; EU417719
G. chinensis	HM802951; HM802952; HM802953; HM802954
G. gecko	JF920685; JF920686; JF920687; JF920688
G. hokouensis	EU417720; EU417721; EU417722
G. japonicus	EU417723; EU417724; EU417725; EU417726; EU417727; EU417728
G. kikuchii	MH274041; MH274042
G. scabridus	HM802946; HM802947; HM802948; HM802949; HM802950
G. subpalmatus	EU417713; EU417714; EU417715
G. swinhonis	EU417709; EU417710; EU417711; EU417712; HM802938;
G. wenxianensis	EU417703; EU417704; EU417705; EU417706; EU417707; EU417708
Hemidactylus dracaenacolus （outgroup）	KU567362
Hemidactylus granti （outgroup）	KU567372
