# Supplementary material for: Phylogenetic Analysis and Genetic Structure of Schlegel’s Japanese Gecko (Gekko japonicus) from China Based on Mitochondrial DNA Sequences
Source: Genes (Basel). 2022 Dec 21;14(1):18. doi: 10.3390/genes14010018 (PMC9858143; doi:10.3390/genes14010018)
Supplement: Supplementary file 1 [file genes-14-00018-s001.zip › Table S3.pdf]

Table S3 Sample information included in each haplotype of the *G. japonicus COI* gene

Haplotype Serial Number	Sample size	Specimen information
H1	233	AHAQ01 AHAQ02 AHAQ03 AHAQ04 AHAQ05 AHAQ08 AHAQ10 AHAQ12 AHLA07 AHWH01 AHWH02 AHWH03 AHWH04 AHWH05 AHWH07 AHWH08 AHWH09 AHWH10 AHWH12 AHWH15 FJNP29 FJYA25 GXGL01 GXGL02 GXGL03 GXGL04 GXGL05 GXGL06 GXGL07 GXGL08 GXGL10 GXGL11 GXGL12 GXGL13 GXGL14 GXGL15 GXGL16 GXGL17 GXGL18 GXGL19 GXLS02 GXLS03 GXLS04 GXLS05 GXLS06 GXLS08 GXLS09 GXLS10 GXLS11 GXLS12 GXLS13 GXLS14 GXYF04 GXYS01 GZGD01 GZGD02 GZHX01 GZLB01 GZLB02 GZLB03 GZLL01 GZLL02 HBJM05 HBWH01 HBWH02 HBWH04 HBWH05 HBWH06 HBWH07 HBWH09 HBWH11 HNCB01 HNCB02 HNCB03 HNCB05 HNCB06 HNCB07 HNCB09 HNCB10 HNCB12 HNCB13 HNCB14 HNCB15 HNCB17 HNCD01 HNCD04 HNCD05 HNCD06 HNCD07 HNCD08 HNCD09 HNDX04 HNDX05 HNDX08 HNDX09 HNDX10 HNDX11 HNDX17 HNDX18 HNDX19 HNDX23 HNDX25 HNHH05 HNHH07 HNHT03 HNHT06 HNHT10 HNHT11 HNHT17 HNHY01 HNHY03 HNHY04 HNHY05 HNHY07 HNHY08 HNHY09 HNHY10 HNHY11 HNHY12 HNHY13 HNHY14 HNHY15 HNHY18 HNSP06 HNSP12 HNSY01 HNSY02 HNSY03 HNSY04 HNTD01 HNTD03 HNTD05 HNTD06 HNTD08 HNXH02 HNXH04 HNXH05 HNXH06 HNXH11 HNXH12 HNXH15 HNXH16 HNXH17 HNXH19 HNXH20 HNXN01 HNXN04 HNXN07 HNXN08 HNYY01 HNYY02 HNYY03 HNYY04 HNYY05 HNYY06 HNYY07 HNYY08 HNYY09 HNYY10 HNYY11 HNYY12 HNYZ02 HNYZ04 HNYZ05 HNYZ07 HNYZ09 HNYZ13 HNYZ14 HNZZ01 HNZZ02 HNZZ05 HNZZ06 HNZZ08 JSRG03 JSRG04 JXLN01 JXLN02 JXLN03 JXLN04 JXLN05 JXLN06 JXLN07 JXLN08 JXLN09 JXLN10 JXLN11 JXLN12 JXLN14 JXLN15 JXLN16 JXLN17 JXLN18 JXLN20 JXLN21 JXLN22 JXLN23 JXLN24 QTYY01 QTYY04 QTYY05 QTYY06 SXYX04 SXYX09 SXYX10 SXYX11 SXYX13 SXYX25 SXYX26 SXYX27 YZLST09 YZLST1 YZLST10 YZLST12 YZLST2 YZLST3 YZLST5

		YZLST6 YZLST7 YZLST8 ZJHZ06 ZJHZ08 ZJHZ09 ZJHZ10 ZJHZ15 ZJHZ17 ZJLS15 ZJZS01 ZJZS02 ZJZS03 ZJZS04 ZJZS07 ZJZS08 ZJZS09
H2	1	AHAQ07
H3	2	AHAQ09 AHAQ11
H4	11	AHWH14 HBWH03 HBWH10 HBWH12 HNTD07 HNYZ15 YZLST11 YZLST4 ZJZS05 ZJZS11 ZJZS12
H5	55	FJYA24 FJYA26 GXGL09 GXYF01 GXYF02 GXYF03 GXYF05 HBWH08 HBWH13 HNDX06 HNDX07 HNDX12 HNDX13 HNDX16 HNDX21 HNDX22 HNHH01 HNHH03 HNHH08 HNHH09 HNHH10 HNHH11 HNHH12 HNHH13 HNHH14 HNHH15 HNHH16 HNHH17 HNHH18 HNHY02 HNHY19 HNSP14 HNXH01 HNXH03 HNXH07 HNXH08 HNXH09 HNXH10 HNXH13 HNXH14 HNXH18 HNYZ01 HNYZ03 HNYZ06 HNYZ10 HNZZ07 QTYY02 SXYX14 SXYX15 SXYX16 SXYX17 SXYX19 SXYX21 SXYX22 ZJHZ16
H6	4	GXLS01 GXLS07 HNDX24 HNZZ04
H7	1	HNCD02
H8	10	HNHT01 HNHT04 HNHT05 HNHT07 HNHT09 HNHT12 HNHT13 HNHT14 HNHT15 HNHT16
H9	1	HNHT08
H10	1	HNSP13
H11	1	HNSY05
H12	2	JXLN13 JXLN19
H13	1	ZJHZ01
H14	1	ZJZS06
