# Supplementary figures and images for: Phylogenetic Analysis and Genetic Structure of Schlegel’s Japanese Gecko (Gekko japonicus) from China Based on Mitochondrial DNA Sequences
Source: Genes (Basel). 2022 Dec 21;14(1):18. doi: 10.3390/genes14010018 (PMC9858143; doi:10.3390/genes14010018)

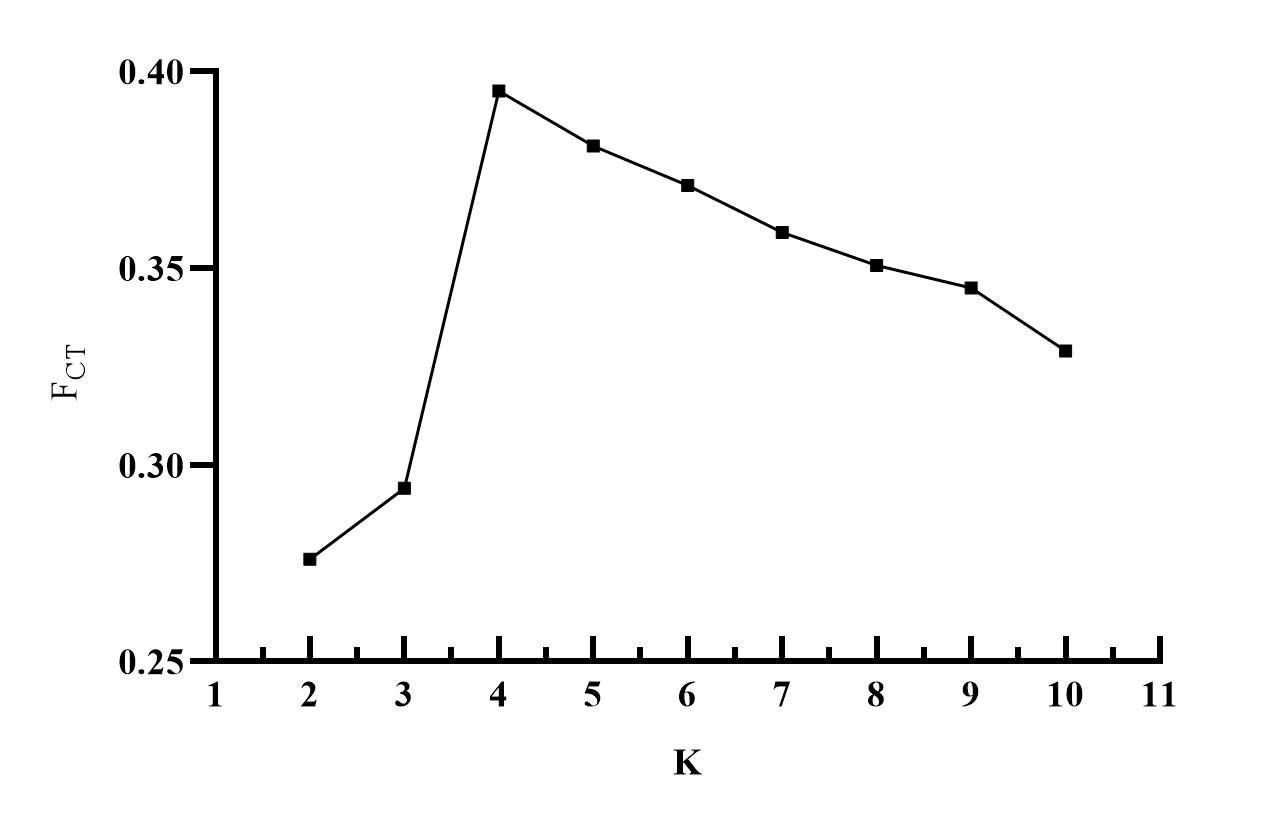

Supplement: Supplementary file 1 [file genes-14-00018-s001.zip › Figure S1.tif]

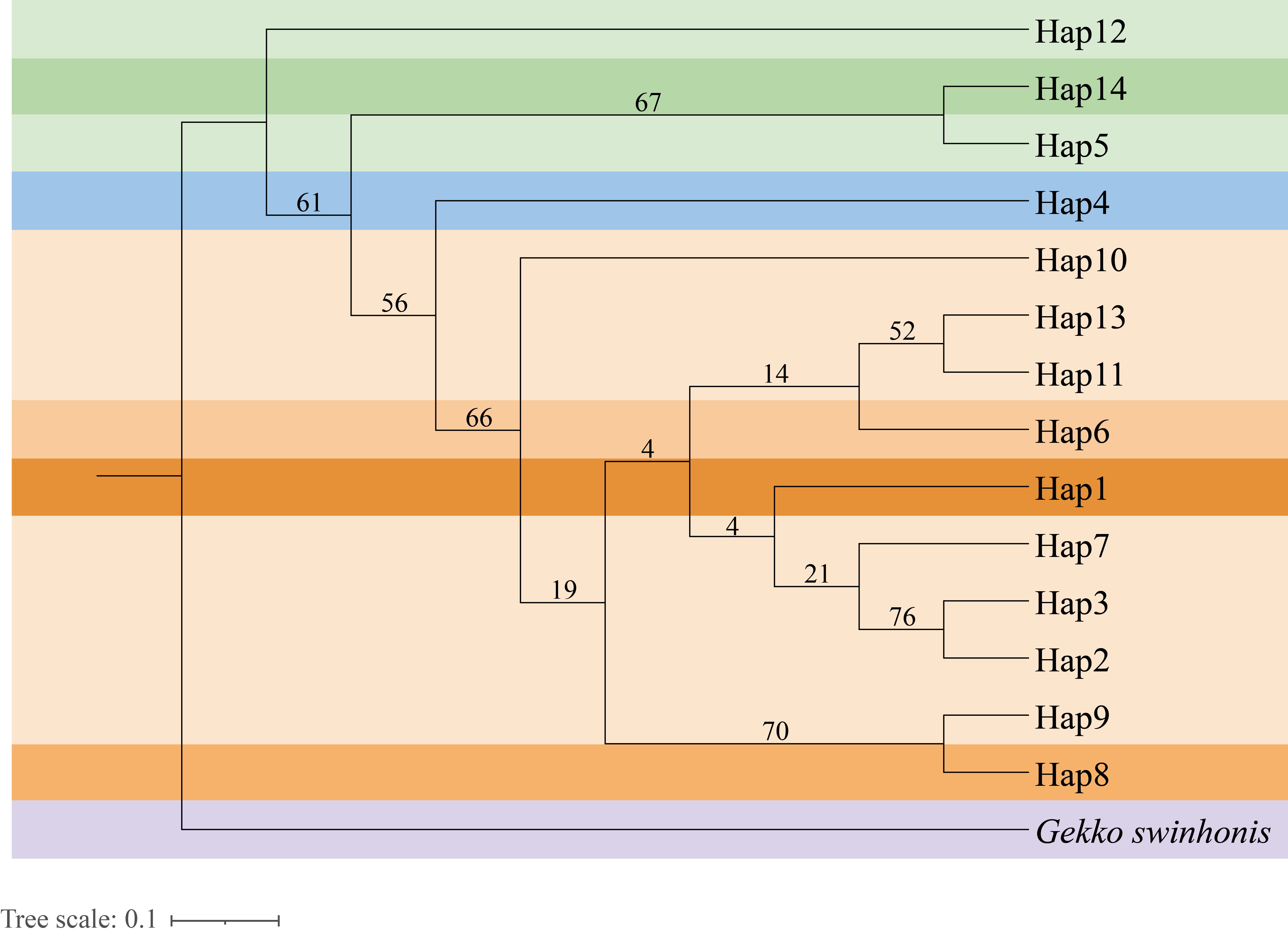

Supplement: Supplementary file 1 [file genes-14-00018-s001.zip › Figure S2.tif]
